# Evaluation of pharmacokinetic interactions between long‐acting cabotegravir or emtricitabine/tenofovir disoproxil fumarate and hormonal contraceptive agents: a tertiary analysis of South African participants in HPTN 084

**DOI:** 10.1002/jia2.70056

**Published:** 2025-10-31

**Authors:** Mark A. Marzinke, Brett Hanscom, Daniel Haines, Kimberly K. Scarsi, Yaw Agyei, Estelle Piwowar‐Manning, Craig W. Hendrix, Ryann Gollings, Scott Rose, Carrie Mathew, Ravindre Panchia, Elizabeth Spooner, Nishanta Singh, Peter Bock, Alex R. Rinehart, Susan L. Ford, James F. Rooney, Lydia Soto‐Torres, Myron S. Cohen, Mina C. Hosseinipour, Sinead Delany‐Moretlwe, HPTN 084 Study Team

**Affiliations:** ^1^ School of Medicine Johns Hopkins University Baltimore Maryland USA; ^2^ Vaccine and Infectious Disease Division Fred Hutchinson Cancer Center Seattle Washington USA; ^3^ College of Pharmacy University of Nebraska Medical Center Omaha Nebraska USA; ^4^ FHI 360 Durham North Carolina USA; ^5^ Wits RHI University of the Witwatersrand Johannesburg South Africa; ^6^ Perinatal HIV Research Unit University of the Witwatersrand Johannesburg South Africa; ^7^ HIV and Other Infectious Diseases Research Unit South African Medical Research Council Durban South Africa; ^8^ Desmond Tutu TB Centre Department of Paediatrics Stellenbosch University Cape Town South Africa; ^9^ ViiV Healthcare Durham North Carolina USA; ^10^ GlaxoSmithKline Research Triangle Park North Carolina USA; ^11^ Gilead Sciences Foster City California USA; ^12^ Division of AIDS National Institute of Allergy and Infectious Diseases Rockville Maryland USA; ^13^ University of North Carolina (UNC) at Chapel Hill Chapel Hill North Carolina USA; ^14^ UNC Project‐Malawi Lilongwe Malawi

**Keywords:** cabotegravir, contraception, HIV prevention, long‐acting, PrEP, women

## Abstract

**Introduction:**

HPTN 084 found that long‐acting cabotegravir (CAB‐LA) was well‐tolerated and significantly reduced the risk of HIV acquisition in women compared to tenofovir disoproxil fumarate/emtricitabine (F/TDF). During the blinded phase of the trial, participants were required to use an effective method of contraception, including an injectable or implantable hormonal contraceptive (HC) agent. A contraceptive sub‐study assessed the pharmacokinetic interactions between pre‐exposure prophylaxis agents (CAB‐LA or F/TDF) and etonogestrel (ENG), medroxyprogesterone acetate (MPA) or norethindrone enanthate (NET‐EN).

**Methods:**

Participants were enrolled in a nested sub‐study between 24 February 2020 and 26 October 2020. Via a convenience sampling strategy, plasma concentrations of ENG, MPA and NET‐EN were evaluated at enrolment and weeks 25, 49 and 73; plasma tenofovir (TFV) and CAB concentrations were determined at contemporaneous visits. Participants were allowed to switch contraceptives, and HC assessments were adjusted accordingly. Geometric mean concentrations were calculated and compared using *t*‐tests or Fisher's exact tests.

**Results:**

One hundred and seventy participants were included in this analysis. Hormone concentrations at all study visits were comparable between the CAB‐LA and F/TDF study arms. Among participants randomized to the CAB‐LA arm, geometric mean concentrations declined from enrolment to the follow‐up period for ENG (335 to 202 pg/ml), MPA (1520 to 1138 pg/ml) and NET‐EN (3715 to 1888 pg/ml); similar findings were observed among participants randomized to the F/TDF arm. Observed HC declines are likely attributed to the timing of contraceptive administration relative to sampling; the percentage of participants with hormone concentrations above thresholds associated with ovulation suppression was high (73−100%) and did not differ between arms. CAB concentrations were comparable across contraceptive types, with 97.8−98.1% of participants yielding trough CAB concentrations above the protocol‐specified target threshold. TFV concentrations were unquantifiable for most participants, irrespective of contraceptive agent, rendering comparisons largely uninformative.

**Conclusions:**

Given the comparable hormone concentrations between arms and the likely influence of the timing of sample collection on observed measurements, clinically significant interactions between CAB‐LA and HC are not expected. Associations between F/TDF and hormone concentrations could not be effectively evaluated due to low adherence to F/TDF.

**Clinical Trial Registration:**

NCT0316456

## 1 INTRODUCTION

HIV remains a public health priority, with several groups who are at high risk of viral acquisition, including women aged 15−49 in Eastern and Southern Africa, who account for more than half of new HIV acquisitions globally [1]. While several biomedical HIV pre‐exposure prophylaxis (PrEP) modalities are efficacious, there is increased interest in long‐acting products for HIV prevention, as they can overcome adherence barriers associated with daily oral regimens observed in key populations, including women [2]. Cabotegravir (CAB) is an integrase strand transfer inhibitor that has been formulated as a long‐acting nanosuspension for intramuscular (IM) administration for the prevention of HIV acquisition [3]. The superiority of long‐acting cabotegravir (CAB‐LA) to oral tenofovir disoproxil fumarate/emtricitabine (F/TDF) in the prevention of HIV among women was demonstrated in the phase 3 HPTN 084 trial [4, 5].

Following early reports of an increased incidence of neural tube defects in women living with HIV during peri‐conceptional exposure to dolutegravir, a structural analogue of CAB, the use of a modern contraceptive method with a failure rate of <2% was incorporated as an HPTN 084 enrolment requirement; participants had access to a range of contraceptives, including injectable and implantable hormonal contraceptive (HC) agents [4]. While subsequent studies demonstrated no increased risk of neural tube defects with dolutegravir exposure, HC use remained in place during the blinded phase of HPTN 084 as an additional safety measure [6, 7].

CAB‐LA exhibits absorption‐limited kinetics, in which the concentration‐time profile is influenced primarily by absorption [8, 9]. While CAB is primarily excreted unchanged via the biliary tract, CAB also undergoes glucuronidation via uridine glucuronosyltransferase (UGT) enzyme isoforms UGT1A1 and UGT1A9 to inactive metabolites and is renally cleared [10]. Based on in vitro and clinical studies, CAB neither induces nor inhibits cytochrome P450 (CYP) or UGT metabolic enzymes and is unlikely to be a perpetrator of clinically meaningful drug−drug interactions [11, 12]. Initial work has evaluated interactions between CAB and HCs. Co‐administration of oral CAB with levonorgestrel and ethinyl oestradiol showed no effect of CAB on contraceptive concentrations; further, CAB pharmacokinetic (PK) parameters were comparable to historic controls [13]. In a secondary analysis of HPTN 077, when compared to women not accessing HCs, there were no significant differences in CAB‐LA PK parameters among women using implantable or injectable HCs [14].

Although current data suggest a low likelihood of clinically significant interactions between CAB‐LA and HCs, additional characterization is warranted. Thus, a drug−drug interaction sub‐study was incorporated into HPTN 084. Herein, we evaluated the PK interactions between CAB‐LA and F/TDF and three HCs commonly used in the region.

## 2 METHODS

### 2.1 Study cohort

HPTN 084 enrolled 3224 women in Eastern and Southern Africa. The design and outcomes of the trial have been described previously [4]. The study dosing regimen included a 5‐week oral lead‐in, in which participants received daily oral CAB or F/TDF and placebo pills. Participants then received an initial IM injection of 600 mg CAB or placebo, a second injection 4 weeks later and injections every 8 weeks thereafter until unblinding. Participants were also supplied with oral F/TDF or placebo pills to be taken daily. The protocol was reviewed and approved by national drug authorities and local research ethics committees in each of the participating sites, and written informed consent was required prior to enrolment in the main HPTN 084 study as well as the HPTN 084 HC sub‐study.

During the blinded phase of the study, participants were required to be on an effective modern form of contraception. Frequent HCs used in the region included etonogestrel (ENG) subdermal implants, depot medroxyprogesterone acetate (MPA) and injectable norethindrone enanthate (NET‐EN). ENG implants are FDA‐approved for 3 years but may remain effective longer [15]. MPA is indicated to be administered as an IM or subcutaneous injectable every 12 weeks, and NET‐EN is delivered intramuscularly every 8 weeks. Post‐administration, median time to maximum concentrations (t_max_) for implantable ENG, depot MPA and injectable NET‐EN are 4, 8 and 6 days, respectively [16, 17, 18]. Contraceptive administration was allowed through direct delivery at study visits or verification of a contraceptive card that included information on the type and date of HC provided; implants could be inserted on site or verified by palpation. There was no effort to synchronize HC dosing with CAB injections or study visits, and participants were allowed to switch HC methods throughout study conduct. For visits occurring after a participant reported HC discontinuation, participants were required to transition to open‐label F/TDF, and data were excluded. The blinded phase of the trial covered the period of 27 November 2017 through 04 November 2020.

As South Africa was the only country where all three methods were routinely available as contraception, South African participants were recruited and consented into the nested contraceptive sub‐study upon enrolment into the blinded phase of HPTN 084. Additional blood samples were provided at enrolment and study weeks 25, 49 and 73, and collected irrespective of HC method; sampling time points were selected as they were predicted to occur at a similar point in the injection cycle for MPA or NET‐EN; as implantable ENG reaches relatively consistent concentrations 4–6 months post‐implantation, the sampling time points were also predicted to be appropriate for ENG measurements. During the blinded phase of the study, participants were selected so that approximately 60 participants using each of the three HC types were enrolled, with approximately 30 participants from each PrEP arm within each HC group. Participants were enrolled in the sub‐study between 24 February 2020 and 26 October 2020.

### 2.2 Drug measurements

For participants randomized to the CAB‐LA arm, plasma CAB concentrations were determined at study enrolment through study week 73. For participants randomized to the F/TDF arm, plasma tenofovir (TFV) and emtricitabine (FTC) concentrations were measured at enrolment, and study weeks 25, 49 and 73; while both compounds were measured, only plasma TFV concentrations were used to interpret adherence [4]. Plasma HC concentrations were evaluated at enrolment, and study weeks 25, 49 and 73, and analysis was performed based on the contraceptive type reported in the period prior to that study visit. For participants who switched HC type during the study, relevant HC concentrations were measured at subsequent visits.

Study drug and HC concentrations were determined via validated liquid chromatographic‐tandem mass spectrometric (LC‐MS/MS) assays as previously described [19, 20, 21]. Analytical measuring ranges were as follows: CAB, 0.25−25.0 µg/ml; TFV, 0.31−1000 ng/ml; FTC, 0.31−5000 ng/ml; ENG, MPA and NET‐EN, 20−10,000 pg/ml.

### 2.3 Interpretation of drug concentrations

The in vitro protein‐adjusted 90% CAB inhibitory concentration (PA‐IC_90_) is 0.166 µg/ml [22, 23]. CAB concentrations were interpreted relative to the protocol‐specific target threshold (4x PA‐IC_90_; 0.664 µg/ml). Plasma TFV concentrations > 40 ng/ml are associated with daily adherence to oral F/TDF [19, 24]. HC measurements do not necessarily reflect trough concentrations, and measurements were contextualized based on the timing of sample collection relative to the last HC dose. Contraceptive concentrations associated with ovulation suppression are greater than or equal to 90 pg/ml for ENG, 100 pg/ml for MPA and 1000 pg/ml for NET‐EN [25, 26, 27, 28].

### 2.4 Statistical analysis

Statistical analysis was limited to all consented participants who provided additional blood samples beyond the enrolment visit. CAB‐LA concentration‐time profiles were generated for participants by each contraceptive type, and geometric mean trough concentrations were compared based on the HC agent used. Geometric mean plasma TFV concentrations were determined at the aforementioned study visits. At each evaluated visit, contraceptive type was categorized, including time from last HC administration; participants who switched their HC regimen during the sub‐study were included in multiple categories. Geometric mean concentrations of ENG, MPA and NET‐EN, and percentages of participants with contraceptive concentrations associated with ovulation suppression were compared between the CAB‐LA and F/TDF study arms at enrolment and study weeks 25, 49 and 73. Comparison of geometric HC concentrations between enrolment (pre‐initiation of CAB‐LA or F/TDF) and post‐enrolment visits was also conducted; geometric means of post‐enrolment HC concentrations were pooled across all observations. Two‐sided *t*‐tests were used for between‐group comparisons, generalized estimating equation (GEE) models were used to compare baseline and follow‐up concentrations, and Fisher's exact tests were used to compare the proportion of participants above the ovulation suppression and PrEP target thresholds; *p*‐values < 0.05 were considered significant.

GEE models were used to evaluate the association between plasma HC and CAB‐LA or TFV concentrations, accounting for multiple observations per participant. ENG concentrations >500 pg/ml (*n* = 2), MPA concentrations >3000 pg/ml (*n* = 2) and NET‐EN concentrations >8000 pg/ml (*n* = 1) were anomalously high and considered outliers based on expected concentrations relative to time of administration and were excluded from analysis [25, 26, 27, 28]. In all GEE models, missing assessments were treated as missing completely at random. All analyses were performed using R (version 4.3.1).

## 3 RESULTS

### 3.1 Demographics

Of the 3224 women enrolled in HPTN 084, 190 participants from South African sites consented to the HC sub‐study. Twenty participants did not participate beyond the enrolment visit, resulting in 170 evaluable participants; 80 were randomized to the CAB‐LA arm and 90 to the F/TDF arm. Across both arms, median age and body mass index (BMI) at enrolment were 22 years (IQR: 20, 25.8 years) and 28.3 kg/m^2^ (IQR: 24.1, 32.4 kg/m^2^), respectively. Median BMI at baseline was slightly higher for participants randomized to the F/TDF study arm (F/TDF: 28.8 kg/m^2^; CAB‐LA: 27.1 kg/m^2^) (Table 1).

**TABLE 1:** Baseline demographics of participants who consented and provided post‐enrolment samples in the hormonal contraceptive sub‐study

	CAB‐LA arm	F/TDF arm	All participants
**Participants, *n***	80	90	170
**Median age, years (IQR)**	23 (21, 26)	22 (20, 25)	22 (20, 2)
18−29 years of age, *n* (%)	73 (91%)	80 (89%)	153 (90%)
30−39 years of age, *n* (%)	6 (8%)	10 (11%)	16 (9.4%)
40−45 years of age, *n* (%)	1 (1%)	0 (0%)	1 (0.6%)
**Median BMI, kg/m^2^ (IQR)**	27.1 (22.8, 31.3)	28.8 (25.2, 32.7)	28.3 (24.1, 32.4)
BMI < 30 kg/m^2^, *n* (%)	54 (68%)	51 (57%)	105 (62%)
BMI ≥ 30 kg/m^2^, *n* (%)	26 (32%)	39 (43%)	65 (38%)
**Baseline HC agent**			
ENG, *n* (%)	28 (35%)	27 (30%)	55 (32%)
MPA, *n* (%)	23 (29%)	29 (32%)	52 (31%)
NET‐EN, *n* (%)	29 (36%)	34 (38%)	63 (37%)
**Change in HC agent through Week 73, *n* (%)**	15 (19%)	24 (27%)	39 (23%)

Abbreviations: BMI, body mass index; CAB‐LA, long‐acting cabotegravir; ENG, etonogestrel; F/TDF, tenofovir disoproxil fumarate/emtricitabine; HC, hormonal contraceptive; IQR, interquartile range; MPA, medroxyprogesterone acetate; NET‐EN, norethindrone enanthate.

Changes in HC methods were common; from enrolment through study week 73, 23% (39/170) of evaluable participants switched their HC at least once. The most common switches occurred between MPA and NET‐EN (62%); of note, MPA stockouts were common in South Africa during this period (Table S1). Four participants experienced multiple HC changes during the evaluable period.

### 3.2 HC concentrations

**FIGURE 1:**
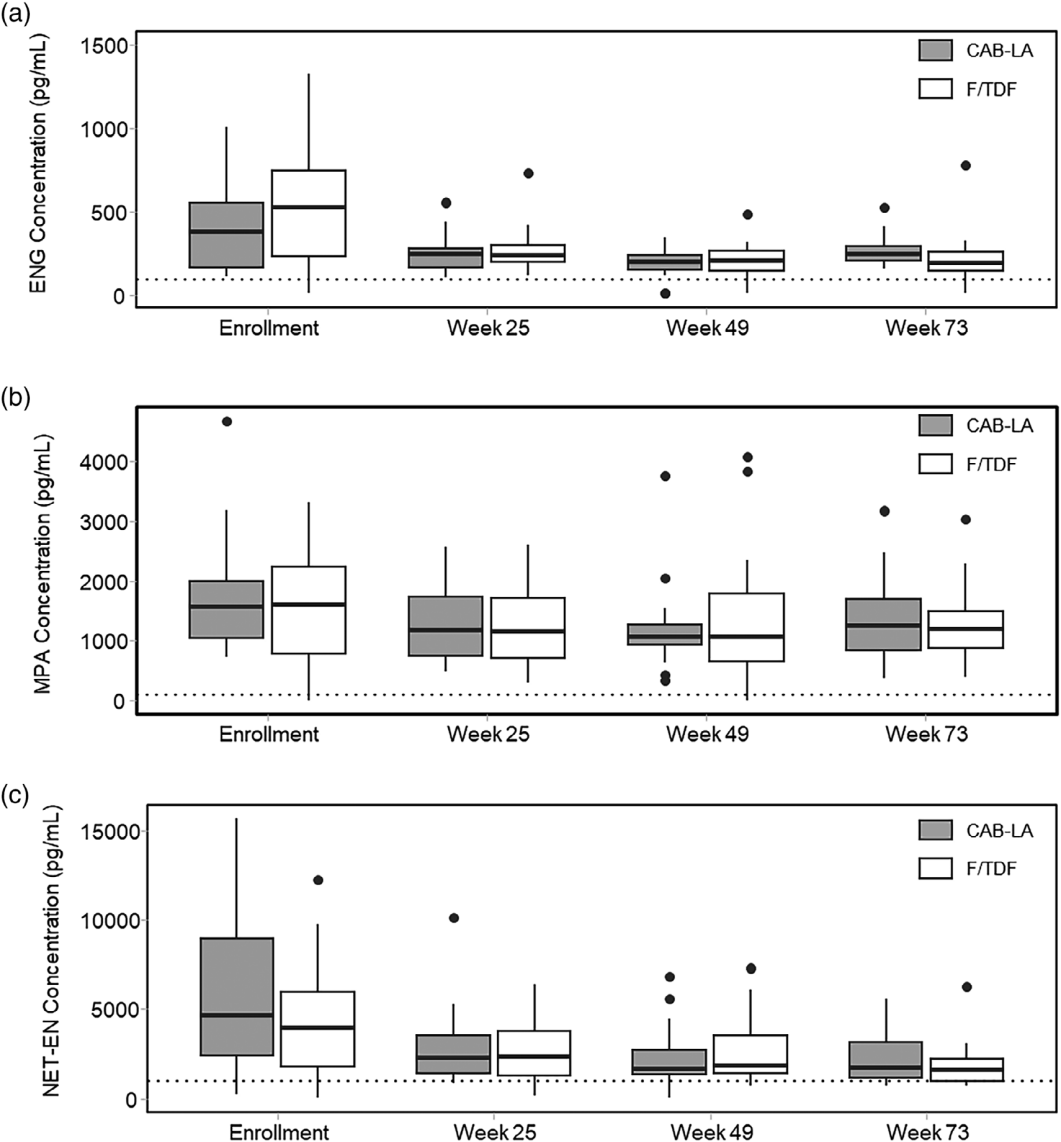
Figure 1. Plasma (a) ENG, (b) MPA and (c) NET‐EN concentrations at enrolment and select post‐enrolment visits. Boxplots of post‐enrolment HC concentrations are pooled across study weeks 25, 49 and 73, stratified by study arm (CAB‐LA or F/TDF). CAB‐LA, long‐acting cabotegravir; ENG, etonogestrel; F/TDF, tenofovir disoproxil fumarate/emtricitabine; HC, hormonal contraceptive; MPA, medroxyprogesterone acetate; NET‐EN, norethindrone enanthate. Dashed lines reflect cutoffs for ovulation suppression (ENG = 90 pg/ml; MPA = 100 pg/ml; NET‐EN = 1000 pg/ml).

HC concentrations were compared between study arms, as well as between enrolment and post‐enrolment visits; visits were not necessarily synchronized with predicted trough contraceptive concentrations. HC concentrations were comparable between study arms at enrolment and study weeks 25, 49 and 73. Geometric mean HC concentrations at enrolment, which reflect contraceptive concentrations before CAB‐LA or F/TDF initiation, were higher than those observed after PrEP initiation (Figure 1). In the CAB‐LA arm, post‐enrolment ENG, MPA and NET‐EN concentrations declined by 40% (*p* = 0.001), 25% (*p* = 0.334) and 49% (*p* = 0.035), respectively; similar trends were observed in the F/TDF arm. As the use of a long‐acting HC was a requirement for inclusion in this study, these observed differences between enrolment and post‐enrolment HC concentrations are attributed to the timing of sample collection relative to HC administration. Sample collection relative to HC administration was more proximate for the enrolment visit than for post‐enrolment visits; geometric mean concentrations and median time from HC administration are summarized in Table 2.

**TABLE 2:** Geometric mean HC concentrations at evaluable study visits and frequency of visits that with ENG, MPA or NET‐EN concentrations associated with ovulation suppression

	CAB‐LA arm			F/TDF arm			
Visit	Geometric mean [HC] pg/ml (95% CI)	Median time from last HC dose, days (IQR)	[HC] above ovulation suppression cutoff, %^a^	Geometric mean [HC] pg/ml (95% CI)	Median time from last HC dose, days (IQR)	[HC] above ovulation suppression cutoff, %^a^	*p*‐value^b^
**ENG**							
Enrolment	335 (261, 430)	34 (11, 498)	100%	390 (237, 642)	12 (8, 84)	93%	0.577
Week 25	226 (193, 264)	210 (184, 657)	100%	247 (210, 291)	189 (186, 216)	100%	0.417
Week 49	158 (110, 227)	371 (351, 776)	92%	185 (136, 253)	360 (348, 382)	96%	0.501
Week 73	253 (211, 304)	532 (517, 551)	100%	174 (101, 301)	522 (509, 531)	93%	0.183
Overall follow‐up	202 (173, 237)	435 (208, 695)	97%	205 (172, 244)	355 (191, 505)	95%	0.924
*p*‐value (enrolment vs. follow‐up)	<0.001	N/A	N/A	<0.001	N/A	N/A	N/A
**MPA**							
Enrolment	1520 (1234, 1873)	34 (12, 44)	100%	1210 (808, 1813)	21 (12, 33)	97%	0.309
Week 25	1174 (964, 1429)	54 (28, 62)	100%	1111 (914. 1350)	54 (28, 78)	100%	0.686
Week 49	1080 (842, 1386)	52 (29, 62)	100%	943 (625, 1424)	54 (34, 71)	97%	0.565
Week 73	1153 (840, 1584)	46 (27, 78)	100%	1124 (905, 1394)	57 (44, 84)	100%	0.886
Overall follow‐up	1138 (996, 1300)	53 (28, 64)	100%	1053 (893, 1242)	56 (36, 84)	98%	0.468
*p*‐value (enrolment vs. follow‐up)	0.334	N/A	N/A	0.238	N/A	N/A	N/A
**NET‐EN**							
Enrolment	3715 (2455, 5620)	11 (8, 16)	86%	2810 (1819, 4343)	10 (9, 21)	91%	0.347
Week 25	2299 (1802, 2934)	28 (27, 45)	92%	1883 (1301, 2724)	32 (23, 56)	79%	0.358
Week 49	1535 (927, 2543)	56 (41, 62)	84%	2193 (1627, 2956)	56 (30, 56)	95%	0.216
Week 73	1891 (1457, 2453)	55 (44, 58)	84%	1544 (1219, 1956)	56 (33, 56)	73%	0.239
Overall follow‐up	1888 (1548, 2302)	50 (28, 57)	87%	1852 (1547, 2217)	49 (28, 56)	85%	0.888
*p*‐value (enrolment vs. follow‐up)	0.035	N/A	N/A	0.977	N/A	N/A	N/A

Abbreviations: CAB‐LA, long‐acting cabotegravir; CI, confidence interval; ENG, etonogestrel; F/TDF, tenofovir disoproxil fumarate/emtricitabine; HC, hormonal contraceptive; IQR, interquartile range; MPA, medroxyprogesterone acetate; NET‐EN, norethindrone enanthate.

^a^ Cutoff concentration associated with ovulation suppression for ENG: ≥ 90 pg/ml; MPA: ≥ 100 pg/ml; NET‐EN ≥ 1000 pg/ml.

^b^ Evaluation of geometric mean hormone concentrations.

As ENG is administered as a subdermal implant, the median time from ENG implantation prior to participant enrolment was 34 days (IQR: 11, 498 days) in the CAB‐LA arm and 12 days (IQR: 8, 84 days) in the F/TDF arm. Geometric mean ENG concentrations at enrolment were 335 pg/ml (95% CI: 261, 430 pg/ml) in the CAB‐LA arm and 390 pg/ml (95% CI: 237, 642 pg/ml) in the F/TDF arm (*p* = 0.577). ENG concentrations were comparable at subsequent visits across study arms. Two participants in the CAB‐LA arm and two in the F/TDF arm had ENG concentrations below the 90 pg/ml cutoff.

At enrolment, geometric mean MPA concentrations were 1520 pg/ml (95% CI: 1234, 1873 pg/ml) in the CAB‐LA arm and 1210 pg/ml (95% CI: 808, 1813 pg/ml) in the F/TDF arm (*p* = 0.309). The median time from MPA injection prior to participant enrolment was 34 days (IQR: 12, 44 days) in the CAB‐LA arm and 21 days (IQR: 12, 33 days) in the F/TDF arm. Pooled post‐enrolment, geometric mean MPA concentrations were 1138 pg/ml (95% CI: 996, 1300 pg/ml) in the CAB‐LA arm and 1053 pg/ml (95% CI: 893, 1242) in the F/TDF arm; median time from MPA administration to post‐enrolment sampling was 53 days (IQR: 28, 64 days) in the CAB‐LA arm and 56 days (IQR: 36, 84 days) in the F/TDF arm. Most participants had MPA concentrations above the 100 pg/ml threshold, with only one in the F/TDF arm falling below that cutoff at week 49.

NET‐EN was administered an average of 2 weeks prior to enrolment; geometric mean plasma NET‐EN concentrations were 3715 pg/ml (95% CI: 2455, 5620 pg/ml) in the CAB‐LA arm and 2810 pg/ml (95% CI: 1819, 4343 pg/ml) in the F/TDF arm (*p* = 0.347). The median period from NET‐EN administration to contraceptive sampling was 50 days (IQR: 28, 57 days) in the CAB‐LA arm and 49 days (IQR: 28, 56 days) in the F/TDF arm. Pooled post‐enrolment, geometric mean NET‐EN concentrations were 1888 pg/ml (95% CI: 1548, 2302 pg/ml) in the CAB‐LA arm and 1852 pg/ml (95% CI: 1547, 2217 pg/ml) in the F/TDF arm (*p* = 0.888). In contrast with our observations for ENG and MPA, NET‐EN concentrations more frequently fell below the ovulation suppression cutoff (1000 pg/ml) [25]. At enrolment, and weeks 25, 49 and 73, 14% (4/29), 8% (2/26), 16% (4/25) and 16% (4/25) had NET‐EN concentrations < 1000 pg/ml in the CAB‐LA arm [25]. Similar findings were observed in the F/TDF arm, and there were no statistical differences in the frequency of participants with NET‐EN concentrations less than 1000 pg/ml across both arms (Table 2).

Two pregnancies occurred in participants randomized to the CAB‐LA arm during the evaluable period. One participant had unquantifiable ENG concentrations at the last evaluated study visit, which was 78 days prior to the pregnancy report date; no other contraceptive data were available for this participant. Given the t_1/2_ of ENG post‐implant removal (∼25 hours), it is postulated that the participant was not using contraceptives in the time proximate to the pregnancy report date. The second participant discontinued ENG 92 days prior to the pregnancy report date, and ENG concentrations 107 days prior to the pregnancy report date were 141 pg/ml. The participant subsequently transitioned to NET‐EN after the pregnancy outcome, which was a spontaneous abortion at <20 weeks. CAB concentrations were as expected for both participants. Observed pregnancies are likely related to HC cessation near the time of the pregnancy report dates.

### 3.3 CAB‐LA and F/TDF drug assessments

**FIGURE 2:**
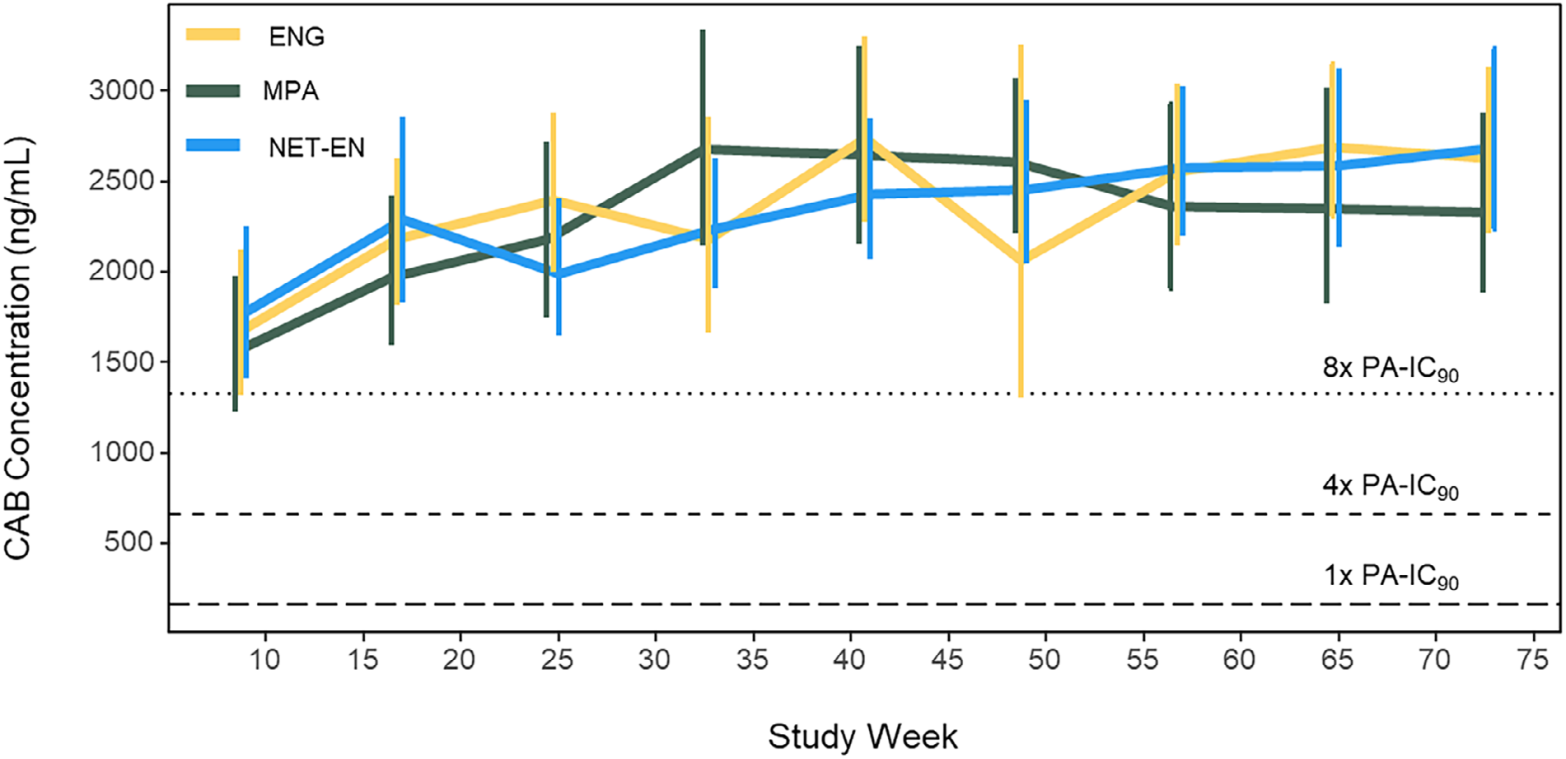
Figure 2. CAB concentration time profiles, stratified by HC agent used. Geometric mean trough CAB concentrations are presented from enrolment through study week 73 and stratified by HC type used. CAB‐LA injections occurred at study weeks 5, 9, 17, 25, 33, 41, 49, 57, 65 and 73. Error bars displayed represent the 95% confidence interval of the geometric mean for each cohort. Yellow: ENG; Olive: MPA; Blue: NET‐EN. CAB, cabotegravir; ENG, etonogestrel; HC, hormonal contraceptive; MPA, medroxyprogesterone acetate; NET‐EN, norethindrone enanthate; PA‐IC_90_, protein‐adjusted 90% inhibitory concentration.

CAB concentration‐time profiles were comparable across all three HC agent groups (Figure 2). There was increased variability in geometric mean CAB concentrations during the oral lead‐in phase, which may be attributed to oral product adherence (data not shown). Over the post‐enrolment period, geometric mean CAB trough concentrations were 2291 ng/ml (95% CI: 2114, 2484 ng/ml) for participants using ENG, 2235 ng/ml (95% CI: 2084, 2397 ng/ml) for participants using MPA and 2305 ng/ml (95% CI: 2171, 2447 ng/ml) for participants using NET‐EN. Across contraceptive types, there were no statistically significant differences in CAB concentrations at any evaluated post‐injection visit, and 98.1% (ENG), 97.8% (MPA) and 97.9% (NET‐EN) of trough CAB‐LA concentrations remained above the protocol‐specific threshold of the 4x PA‐IC_90_ (Table 3).

**TABLE 3:** Geometric mean injection‐phase trough CAB‐LA concentrations in hormonal contraceptive sub‐study participants, randomized to the CAB‐LA study arm and stratified by HC agent

	ENG HC use	MPA HC use	NET‐EN HC use
Geometric mean trough CAB, ng/ml (95% CI)^a^	2291 (2114, 2484)	2235 (2084, 2397)	2305 (2171, 2447)
CAB concentrations >1x PA‐IC_90_ *n* (%)	207/208 (99.5%)	178/178 (100%)	236/236 (100%)
CAB concentrations >4x PA‐IC_90_ *n* (%)	204/208 (98.1%)	174/178 (97.8%)	231/236 (97.9%)
CAB concentrations ≥8x PA‐IC_90_ *n* (%)	188/208 (90.4%)	155/178 (87.1%)	213/236 (90.3%)

Abbreviations: CAB, cabotegravir; CI, confidence interval; ENG, etonogestrel; HC, hormonal contraceptive; MPA, medroxyprogesterone acetate; NET‐EN, norethindrone enanthate; PA‐IC_90_, in vitro protein‐adjusted 90% CAB inhibitory concentration.

^a^ Geometric mean concentrations based on CAB‐LA concentrations observed as injection‐phase trough visits (study weeks 9, 17, 25, 33, 41, 49, 57, 65 and 73).

For participants randomized to the F/TDF arm, plasma TFV concentrations were generally low at all evaluated visits, regardless of HC type. Geometric mean TFV concentrations were 0.50 ng/ml (95% CI: 0.31, 0.83 ng/ml) for participants using ENG, 0.75 ng/ml (95% CI: 0.46, 1.23 ng/ml) for participants using MPA and 0.52 ng/ml (95% CI: 0.32, 0.83 ng/ml) for those using NET‐EN; 65.6% (59/90) of participants in the F/TDF arm had unquantifiable concentrations at all evaluated study visits, irrespective of the HC used (Table 4 and Figure S1). Comparisons of plasma TFV concentrations by HC agents were uninformative, as adherence was poor among participants evaluated in the sub‐study. Only 34.4% (31/90) of participants had at least one plasma TFV measurement greater than 40 ng/ml at any visit, and only seven (7.8%) participants had concentrations above 40 ng/ml at all evaluable post‐enrolment visits. There were no instances of HIV acquisition among participants randomized to the CAB‐LA arm during the evaluation period; four HC sub‐study participants randomized to the F/TDF arm acquired HIV during the evaluable period; however, adherence to oral F/TDF was poor among this cohort.

**TABLE 4:** Geometric mean plasma TFV concentrations in hormonal contraceptive sub‐study participants, randomized to the F/TDF study arm and stratified by HC agent

Study visit	ENG HC use	MPA HC use	NET‐EN HC use
Enrolment TFV, ng/ml (95% CI)	0.16^a^ (0.16, 0.16)	0.16^a^ (0.16, 0.16)	0.16^a^ (0.15, 0.18)
Week 25 TFV, ng/ml (95% CI)	0.90 (0.27, 2.98)	2.48 (0.83, 7.36)	0.62 (0.23, 1.63)
Week 49 TFV, ng/ml (95% CI)	0.51 (0.19, 1.36)	0.81 (0.28, 2.30)	1.32 (0.36, 4.82)
Week 73 TFV, ng/ml (95% CI)	1.73 (0.32, 9.45)	0.94 (0.28, 3.16)	0.97 (0.25, 3.80)

Abbreviations: CI, confidence interval; ENG, etonogestrel; HC, hormonal contraceptive; MPA, medroxyprogesterone acetate; NET‐EN, norethindrone enanthate; TFV, tenofovir.

^a^ TFV results below the lower limit of quantitation (< 0.31 ng/ml) were designated as 0.16 ng/ml (50% of the lower limit of quantitation).

### 3.4 Association between CAB‐LA and HCs

**FIGURE 3:**
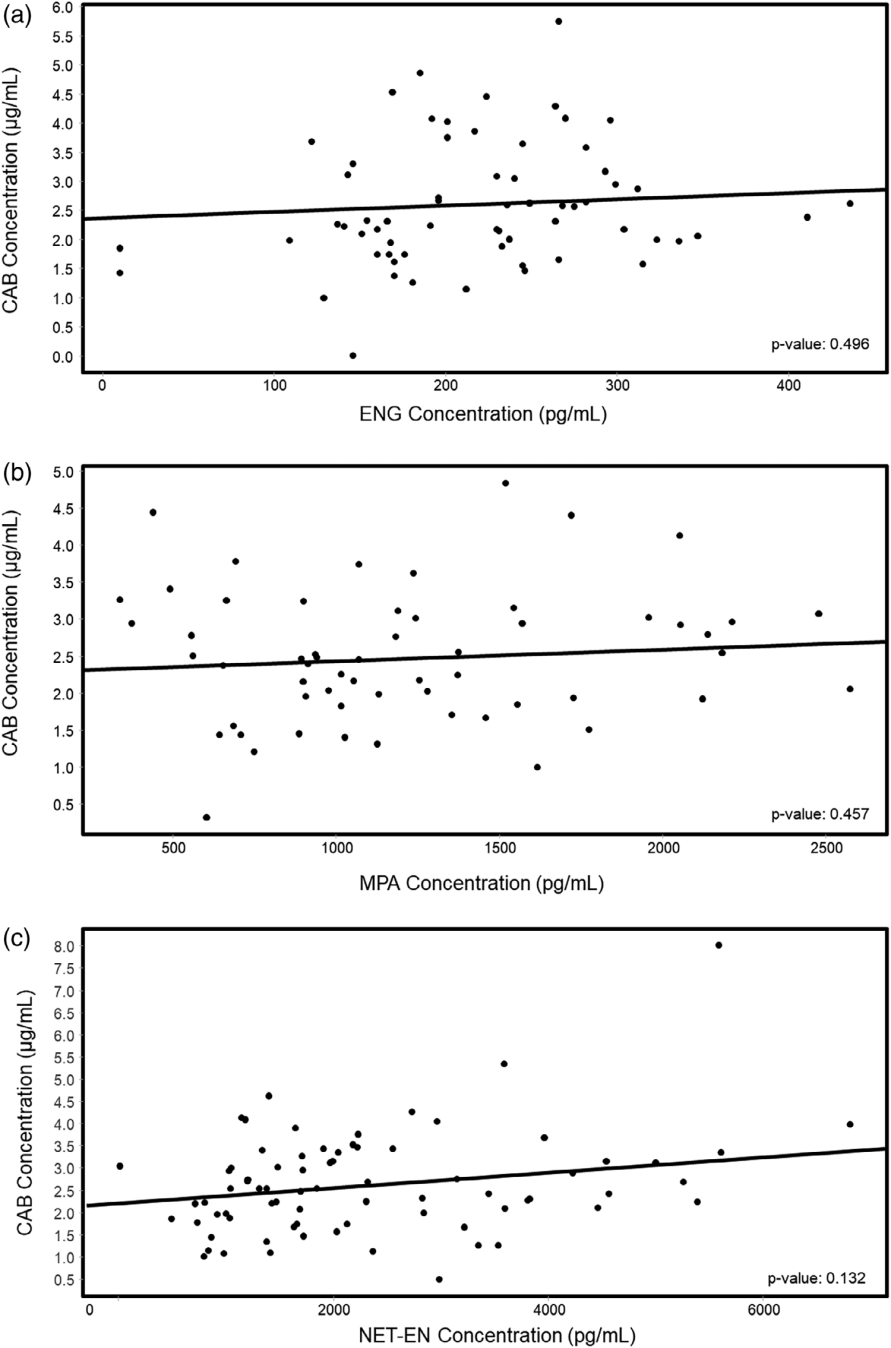
Observed CAB versus HC concentration scatter plots, overlaid with fitted linear regression lines, excluding HC outlier concentrations. After removal of outlier measurements, none of the associations between CAB and HC concentrations were significant. CAB, cabotegravir; ENG, etonogestrel; HC, hormonal contraceptive; MPA, medroxyprogesterone acetate; NET‐EN, norethindrone enanthate.

After the removal of outlier concentrations, none of the statistical models evaluating the association between HC and trough CAB concentrations were significant (Figure 3). Associations between plasma TFV and HC concentrations were not informative given the frequent occurrence of unquantifiable TFV measurements (Figure S2).

## 4 DISCUSSION

In this tertiary analysis of HPTN 084, CAB‐LA concentrationtime profiles were comparable regardless of contraceptive type evaluated, and geometric mean CAB‐LA trough concentrations largely remained above the protocol‐specified target concentration during the injection phase of the study. Associations between HCs and F/TDF could not be robustly evaluated due to low F/TDF adherence in participants enrolled in the sub‐study. While hormone measurements were not synchronized to evaluate trough HC measurements, concentrations of the progestins ENG, MPA and NET‐EN did not differ between the CAB‐LA and F/TDF study arms at the evaluated time points. The pregnancies that occurred within this cohort are attributed to HC cessation proximate to the pregnancy report date and were not attributed to a clinically meaningful drug−drug interaction. These data are consistent with previous findings with other HCs and CAB [13, 14], and provide reassurance that concomitant use of ENG, MPA or NET‐EN and CAB‐LA is not expected to compromise effectiveness.

Oestrogen, progestin or combined oestrogen‐progestin HCs are an important component of family planning; of the 1.2 billion women globally who have a need for family planning, 76% use a non‐hormonal (e.g. sterilization, condoms) or HC method [29, 30]. However, there are geographic differences in contraceptive method preferences globally. Among women aged 15–49, contraceptive pills (27.2%) are the most common contraceptive forms used in the United States; conversely, in Eastern and Southern Africa, non‐oral products, such as injectables and implants (34.1%), are the most commonly utilized modality for family planning needs [31]. Although traditional or modern contraceptives are accessed by a large portion of reproductive‐age women, approximately 43 million adolescent girls and young women have unmet needs for pregnancy prevention tools, which may be driven by a variety of social and structural barriers [31, 32].

Historically, changes in HC type are common. In an evaluation of HC use in South Africa in 2013, it was reported that 41% of adolescent girls underwent at least one HC change [33]. Among participants included in our sub‐study analysis, HC switches were also common, with nearly one quarter (24%) of participants changing their contraceptive type during the evaluable period. While changes were largely attributed to local stockouts of MPA, participant preferences may have also informed switches.

Variability in contraceptive concentrations was observed among sub‐study participants in both the F/DTF and CAB‐LA arms. The observed decline in HC concentrations from enrolment to post‐initiation of CAB‐LA or F/TDF is attributed to sample timing relative to HC administration. Of note, the HPTN 084 contraceptive sub‐study relied on the protocol‐specified visit schedule, and visits were chosen based on the prediction that they would be at a similar point in the injection cycle for MPA or NET‐EN, assuming a 12‐ or 8‐week injection schedule, respectively. On average, while MPA was administered 2 months prior to the enrolment visit among evaluated participants, NET‐EN was administered 2 weeks prior to study enrolment. Consequently, assuming high adherence to each HC regimen, there are more trough concentrations in the utilized sampling strategy for NET‐EN than for MPA; this may explain why NET‐EN concentrations more frequently fell below the posited ovulation suppression threshold of 1000 pg/ml across both study arms [25]. Previous studies have shown high inter‐individual variability in NET‐EN PK parameters, which are influenced by tissue blood flow through the site of injection and the rate of hydrolysis to the active progestin [34]. Previous reports suggest variable NET‐EN bioavailability, ranging from 92 to 202 ng/ml per day, which can influence elimination half‐life and maximum HC concentrations [35].

While prior work has reported significant drug−drug interactions with older antiretroviral drug classes, which has largely been attributed to metabolic enzyme activity modulation, meaningful interactions between HCs and CAB are unlikely because the evaluated HCs do not induce UGT activity, and CAB neither induces nor inhibits CYP3A4, which is the primary metabolic pathway for the evaluated HCs [36, 37]. With regard to F/TDF, we previously reported that MPA does not impact F/TDF PK or renal function [38]. Within this analysis, due to F/TDF non‐adherence, meaningful assessment of the association between F/TDF and HCs could not be determined. These data underscore adherence barriers to oral products and further support the use of long‐acting agents for PrEP.

There are limitations to these analyses. Within HPTN 084, specimen collection events were relatively sparse, and the sub‐study was not associated with additional visits and was not intended to measure trough HC concentrations; rather, it relied on the main study schedule of events, which was designed to evaluate PrEP safety and efficacy while minimizing participant burden. A formal clinical drug interaction study, as per FDA guidance for industry recommendations, was not performed [39]. While canonical drug interaction studies focus more on maximum contraceptive concentrations (C_max_) and the area under the concentration time curve, HPTN 084 was designed to evaluate CAB‐LA efficacy. Although greater PK resolution would be achieved with a formal drug−drug interaction study, the incorporation of these assessments within the Phase 3 HPTN 084 trial allowed us to interrogate several HCs in the background of different HIV PrEP agents.

## 5 CONCLUSIONS

Given the comparable hormone concentrations between arms and the likely influence of the timing of sample collection on observed measurements, clinically significant interactions between CAB‐LA and HC are not expected. Geometric mean trough CAB‐LA concentrations were consistently high, with similar observations from populations not using HCs, and observed contraceptive measurements were consistent with those associated with ovulation suppression. Observed pregnancies do not appear to be attributed to contraceptive failure. Evaluation of drug interactions between HCs and F/TDF could not be assessed robustly due to low oral PrEP adherence among the evaluated cohort.

## COMPETING INTERESTS

None of the authors has a commercial or other association that might pose a conflict of interest, with the following exceptions: SLF is an employee and stockholder of GSK. ARR is an employee of ViiV Healthcare and holds stock of GSK. JFR is an employee and stockholder of Gilead Sciences. CWH holds several patents and related royalties related to specific topical microbicides for PrEP and is a founder of Prionde Biopharma, LLC, a PrEP microbicide company; all conflicts are managed by Johns Hopkins University and HPTN.

## AUTHOR CONTRIBUTIONS

All authors participated in the study, contributed to manuscript preparation and provided critical comments on the final manuscript. MAM, SD‐M, BH, CWH, MCH and MSC designed this analysis; CM, RP, ES, NS and PB supervised protocol implementation with support from SR. MAM, YA, EP‐M, CWH, RG and SLF provided laboratory support and interpretation; KKS provided data interpretation; DH and BH provided data management and statistical support; JFR and ARR provided pharmaceutical support; and LS‐T provided sponsor oversight.

## FUNDING

This study was made possible through funding support from the National Institute of Allergy and Infectious Diseases, Office of the Director, National Institutes of Health (NIH), National Institute on Drug Abuse, and the National Institute of Mental Health, under award numbers UM1AI068619 (HPTN Leadership and Operations Center), UM1AI068617 (HPTN Statistical and Data Management Center) and UM1AI068613 (HPTN Laboratory Center). Additional funding was provided by the Bill & Melinda Gates Foundation (OPP1154174) and ViiV Healthcare. Pharmaceutical support was provided by ViiV Healthcare and Gilead Sciences. The funding source played no role in the data collection or analysis of this manuscript. The authors had final responsibility for the decision to submit for publication.

## DISCLAIMER

The content is solely the responsibility of the authors and does not necessarily represent the official views of the National Institutes of Health.

## ETHICAL CONSIDERATIONS

Written informed consent was obtained from all participants in HPTN 084. The HPTN 084 study protocol was reviewed and approved by the institutional review boards and/or ethics committees and Ministries of Health at participating study sites. Study sites agreed to conduct the study in compliance with United States (US) Health and Human Service regulations (45 CFR 46); applicable US Food and Drug Administration regulations; standards of the International Conference on Harmonization Guideline for Good Clinical Practice (E6); Institutional Review Board/Ethics Committee determinations; all applicable in‐country, state, and local laws and regulations; and other applicable requirements (e.g. US National Institutes of Health, Division of AIDS) and institutional policies.

## Supporting information




**Table S1**. Hormonal contraceptive (HC) switches during the evaluable period among participants included in the HC sub‐study.
**Figure S1**. Plasma TFV concentrations at select post‐enrolment visits. Boxplots of post‐enrolment TFV concentrations are pooled across study weeks 25, 49 and 73, stratified by reported HC type (ENG, MPA, NET‐EN). ENG, etonogestrel; MPA, medroxyprogesterone acetate; NET‐EN, norethindrone enanthate; TFV, tenofovir. Dashed lines reflect cutoff associated with daily oral adherence (plasma TFV = 40 ng/ml).
**Figure S2**. Observed plasma TFV versus HC concentration scatter plots. ENG, etonogestrel; HC, hormonal contraceptive; MPA, medroxyprogesterone acetate; NET‐EN, norethindrone enanthate; TFV, tenofovir.

## Data Availability

Data collected for this study may be made available on request. Data archive will be held at Fred Hutch Cancer Center, Seattle, WA. Requests can be sent to HPTN‐Data‐Access@scharp.org.
