# Supplementary material for: Evaluation of pharmacokinetic interactions between long‐acting cabotegravir or emtricitabine/tenofovir disoproxil fumarate and hormonal contraceptive agents: a tertiary analysis of South African participants in HPTN 084
Source: J Int AIDS Soc. 2025 Oct 31;28(11):e70056. doi: 10.1002/jia2.70056 (PMC12576019; doi:10.1002/jia2.70056)

**Supplementary Table 1.** Hormonal contraceptive (HC) switches during the evaluable period among participants included in the HC sub-study.

|  | **CAB-LA Arm** | | **F/TDF Arm** | | **All Participants** | |  |  |
| --- | --- | --- | --- | --- | --- | --- | --- | --- |
| **Participants, n** | 15 | | 24 | | 39 | |  |  |
| ENG to MPA, n (%) | | | 2 (13.3%) | | 3 (12.5%) | | 5 (12.8%) | |
| ENG to NET-EN, n (%) | | | 2 (13.3%) | | 3 (12.5%) | | 5 (12.8%) | |
| MPA to ENG, n (%) | | | 0 (0.0%) | | 0 (0.0%) | | 0 (0.0%) | |
| MPA to NET-EN, n (%) | | | 4 (26.7%) | | 8 (33.3%) | | 12 (30.8%) | |
| NET-EN to ENG, n (%) | | | 1 (6.67%) | | 0 (0.0%) | | 1 (2.6%) | |
| NET-EN to MPA, n (%) | | | 4 (26.7%) | | 8 (33.3%) | | 12 (30.8%) | |
| Multiple HC Switches, n (%) | | | 2^a^ (13.3%) | | 2^b^ (8.33%) | | 4 (10.3%) | |

^a^One participant switched from NET-EN to MPA and back to NET-EN; the second participant switched from ENG to MPA to NET-EN during the evaluable period. ^b^One participant switched from ENG to MPA to ENG; the second participant switched from MPA to NET-EN to MPA and then back to NET-EN. BMI: body mass index; CAB-LA: long-acting cabotegravir; F/TDF: tenofovir disoproxil fumarate/emtricitabine; ENG: etonogestrel; HC: hormonal contraceptive; MPA: medroxyprogesterone acetate; NET-EN: norethindrone enanthate

**Supplementary Figure 1.** Plasma TFV concentrations at select post-enrollment visits. Boxplots of post-enrollment TFV concentrations are pooled across study weeks 25, 49, and 73, stratified by reported HC type (ENG, MPA, NET-EN). ENG: etonogestrel; MPA: medroxyprogesterone acetate; NET-EN: norethindrone enanthate; TFV: tenofovir. Dashed line reflect cutoff associated with daily oral adherence (plasma TFV = 40 ng/mL).


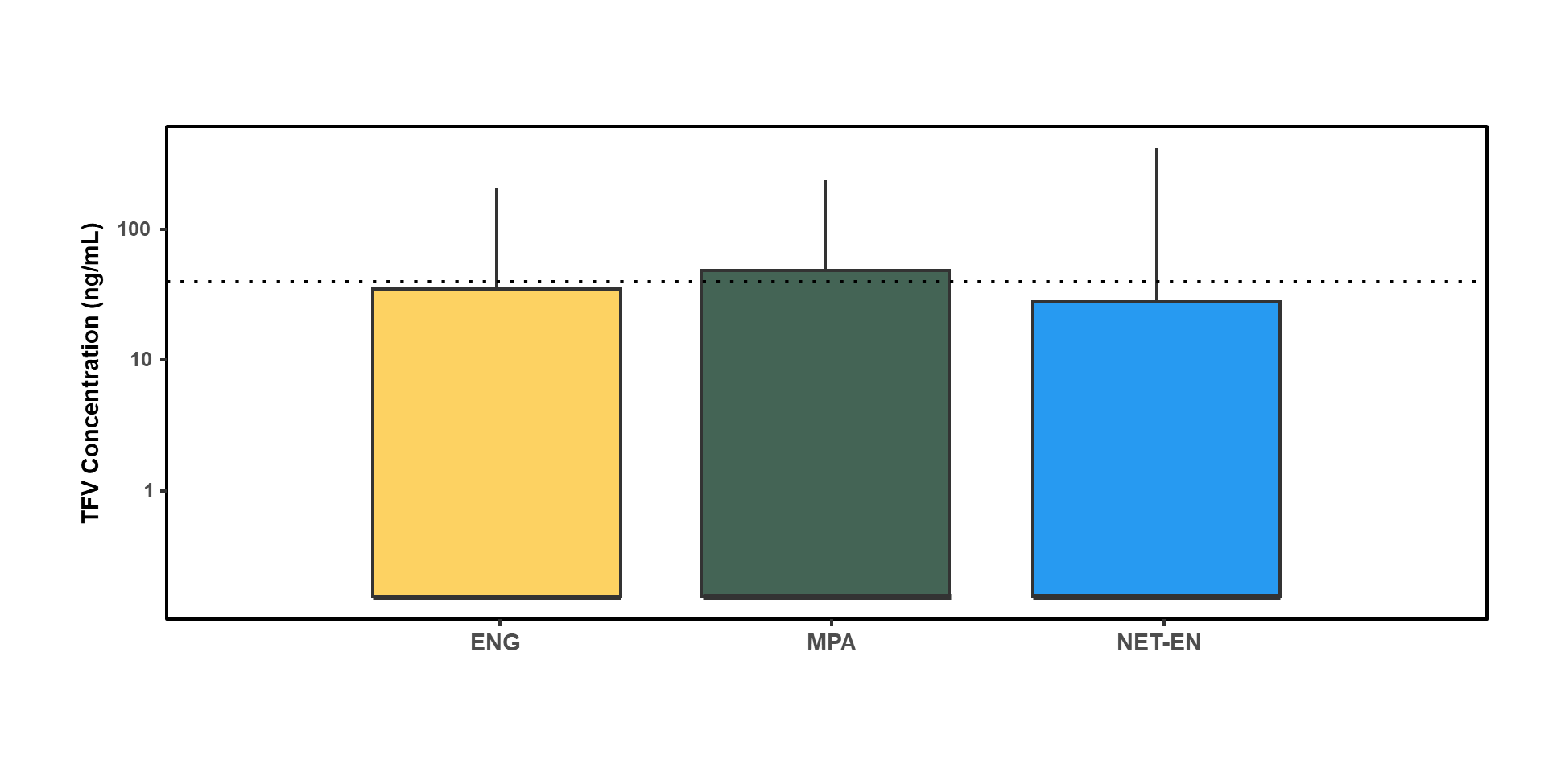


**Supplemental Figure 2.** Observed plasma TFV versus HC concentration scatter plots. HC: hormonal contraceptive; ENG: etonogestrel; MPA: medroxyprogesterone acetate; NET-EN: norethindrone enanthate; TFV: tenofovir.


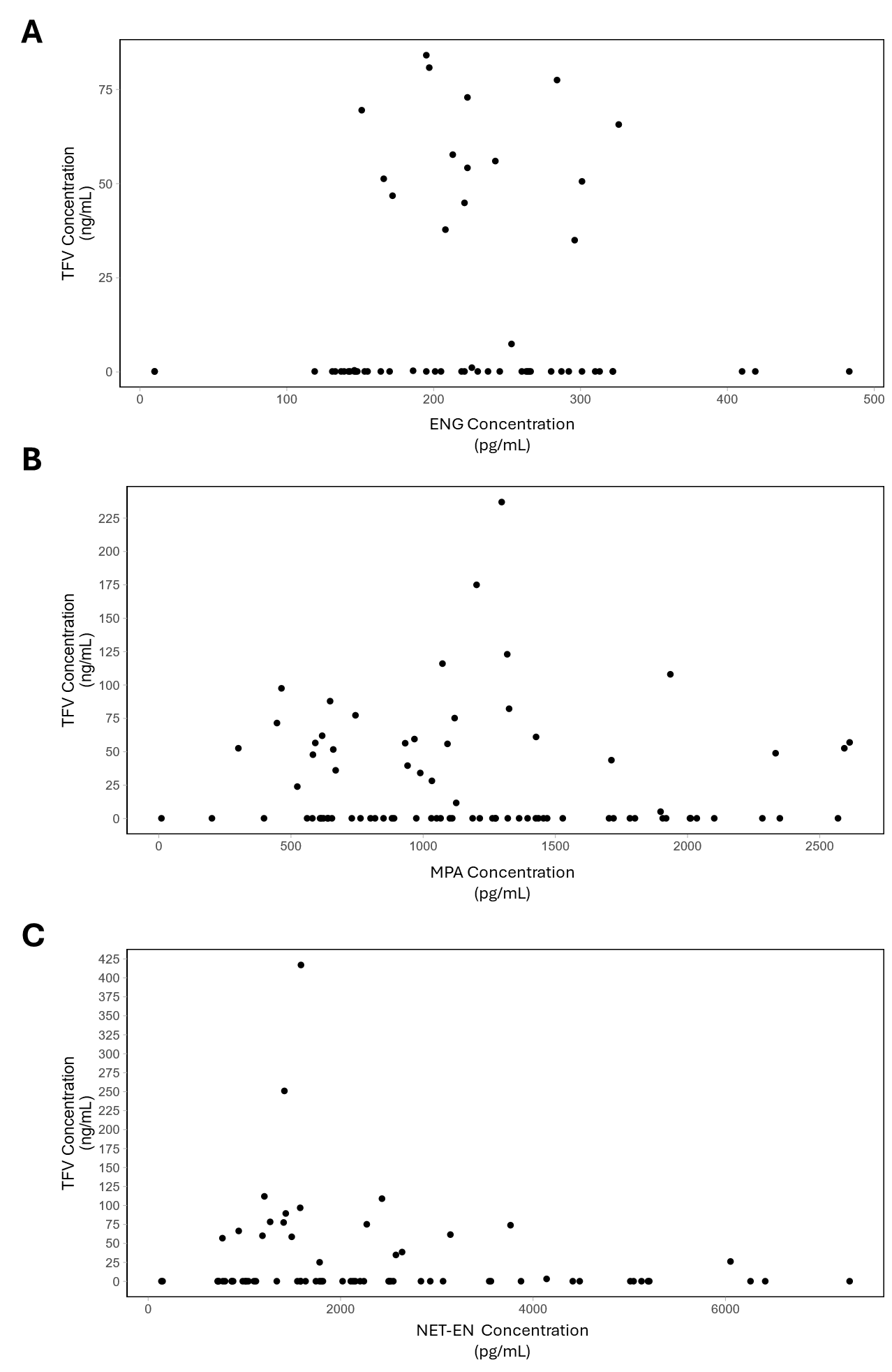

Supplement: Supplementary file 1 — Table S1. Hormonal contraceptive (HC) switches during the evaluable period among participants included in the HC sub‐study. Figure S1. Plasma TFV concentrations at select post‐enrolment visits. Boxplots of post‐enrolment TFV concentrations are pooled across study weeks 25, 49 and 73, stratified by reported HC type (ENG, MPA, NET‐EN). ENG, etonogestrel; MPA, medroxyprogesterone acetate; NET‐EN, norethindrone enanthate; TFV, tenofovir. Dashed lines reflect cutoff associated with daily oral adherence (plasma TFV = 40 ng/ml). Figure S2. Observed plasma TFV versus HC concentration scatter plots. ENG, etonogestrel; HC, hormonal contraceptive; MPA, medroxyprogesterone acetate; NET‐EN, norethindrone enanthate; TFV, tenofovir. [file JIA2-28-e70056-s001.docx]
